# RAPID-DASH: fast and efficient assembly of guide RNA arrays for multiplexed CRISPR-Cas9 applications

**DOI:** 10.1093/synbio/ysaf020

**Published:** 2025-12-18

**Authors:** Asfar Lathif Salaudeen, Nicholas Mateyko, Carl G de Boer

**Affiliations:** Genome Science and Technology Graduate Program, University of British Columbia, Vancouver, British Columbia V6T 1Z4, Canada; Genome Science and Technology Graduate Program, University of British Columbia, Vancouver, British Columbia V6T 1Z4, Canada; School of Biomedical Engineering, University of British Columbia, Vancouver, British Columbia V6T 1Z3, Canada

**Keywords:** CRISPR-Cas9, gRNA array, multiplexing, polymerase cycling assembly, Golden Gate assembly, genome engineering

## Abstract

Guide RNA (gRNA) arrays can enable targeting multiple genomic loci simultaneously using CRISPR-Cas9. In this study, we present a streamlined and efficient method to rapidly construct gRNA arrays with up to 10 gRNA units in a single day. We demonstrate that gRNA arrays maintain robust functional activity across all positions, and can incorporate libraries of gRNAs, combining scalability and multiplexing. Our approach will streamline combinatorial perturbation research by enabling the economical and rapid construction, testing, and iteration of gRNA arrays. To facilitate the adoption of this approach, we have made a web tool to design oligo sequences necessary to assemble gRNA arrays.

## Graphical Abstract

**Graphical Abstract:**
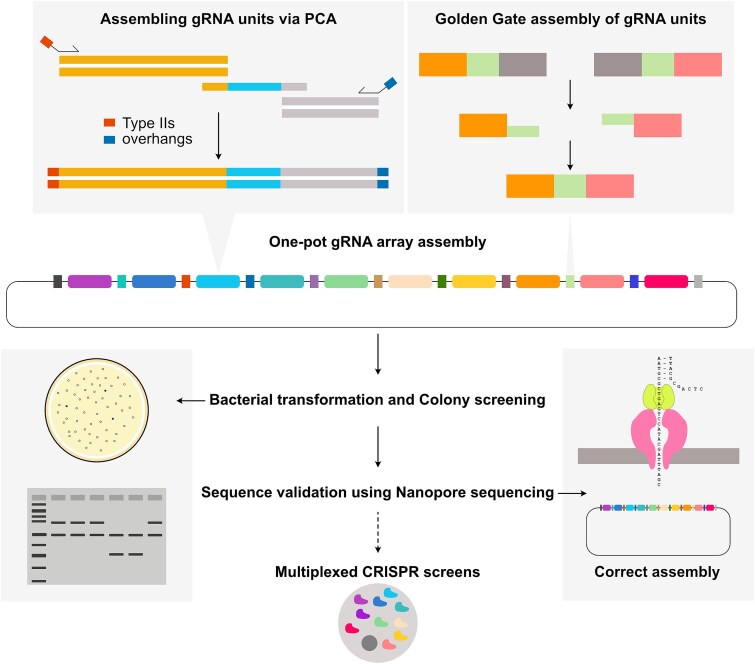

## Introduction

The CRISPR-Cas9 system has revolutionized the field of genome engineering by enabling targeted modification of specific sequences. This system is typically divided into two components: the nuclease protein (Cas9) and a guide RNA (gRNA) that directs Cas9 to its target region [1, 2]. The spacer sequence of the gRNA can be customized to target regions of interest [3]. The nuclease domain has been customized in a variety of ways for different applications, including gene knockouts/knock-ins through double-stranded breaks [4–6], transcriptional regulation (CRISPRa [7] and CRISPRi [8]), and rewriting the genome (base editors [9] and prime editors [10]) and epigenome (CRISPRoff and CRISPRon) [11]. Concurrent delivery of multiple gRNAs is essential to target multiple regions within a cell simultaneously [12]. For instance, Perturb-seq screens target multiple genes within a single cell to identify genetic interactions and reveal cellular pathways [13]; lineage tracing and event recording often edit multiple recorder modules simultaneously to record the history of a cell [14–16]; and targeting the same gene with multiple gRNAs can increase the effect of CRISPRi and CRISPRa in pooled CRISPR-screens [17].

Despite the numerous applications requiring gRNA multiplexing, concurrent cloning and delivery of multiple guides still faces several challenges. An ideal solution would ensure that a single construct includes all the gRNAs of interest to prevent different cells getting different amounts of each gRNA, as would happen with delivery of individually cloned gRNAs. For transfection, this may also be more efficient as, for the same mass of DNA, the concentration and thus the activity of each guide is higher when cloned on the same plasmid as opposed to transfecting individually pooled gRNA expressing plasmids. Integrating guides into the genome is often required for sustained expression and consistent dosage across cells; here too, having all guides on a single construct can facilitate their simultaneous integration because only a single selectable marker is needed to ensure successful delivery. Several approaches have been reported to build multiplexed gRNA systems [12] (Table 1). Polycistronic pre-gRNAs can be processed into functioning gRNAs by simultaneous expression of the Csy4 nuclease [19, 20, 24]. However, this approach requires co-expression of exogenous Cys4 ribonuclease and gRNAs positioned towards the end of the long RNA polymerase III-transcribed polycistronic array were not functional [20]. It is also possible to leverage endogenous tRNA processing by interspersing gRNA units with tRNA sequences in a polycistronic pre-gRNA [21, 22, 25, 26], but this can perturb endogenous tRNA pools. Another technique involves creating arrays of self-contained gRNA units using Gibson assembly, but this approach tends to be inefficient, especially for many gRNA units, resulting in incomplete assemblies. This approach also requires multiple long primers for every gRNA unit as the spacer sequences act as the homology arms to mediate Gibson assembly [23]. Another study used Golden Gate assembly of cloned gRNA fragments into a single vector, including up to 10 gRNAs total, but the initial gRNA cloning and sequence verification adds substantially to the total cloning time and cost [18].

**Table 1:** Summary of RAPID-DASH compared with other gRNA array approaches

**Study**	**Assembly approach**	**Maximum gRNAs assembled**	**gRNA processing method**	**Overall assembly time**	**Pre-cloning step**	**Scalability(a)**	**Oligos per gRNA (length)**	**Other limitations**
RAPID-DASH	Golden Gate assembly	10 gRNAs (up to 20 gRNA units can be assembled using RAPID-DASH online tool)	Individual expression cassettes	3 days	No	Scalable as gRNA oligos can be pooled during PCA to assemble gRNA array libraries.	1 (59 bp)	Long read sequencing required to screen clones.
Vad-Nielsen *et al.* [18]	Golden Gate assembly	30 gRNAs (Assembled in 2 stages)	Individual expression cassettes	>7 days	Yes	Cloning each gRNA restricts scalability.	2 (25 bp)	Labour-intensive: multiple rounds of cloning, culturing and sequence validation.
McCarty *et al.* [19]	Golden Gate assembly	12 gRNAs	Csy4 processing	>2 days	No	Scalable as the gRNA units can be pooled before adding type IIS overhangs for the array assembly (Not demonstrated in the study).	1 (53 bp)	Labour-intensive: multiple rounds of PCR, digestion, and gel purifications. Requires Csy4 endonuclease for individual gRNA processing.
Kurata *et al.* [20]	Golden Gate assembly	10 gRNAs	Csy4 processing	>5 days	Yes	Cloning each gRNA restricts scalability.	2 (25 bp)	Labour-intensive: multiple rounds of cloning, culturing, and sequence validation. Csy4 endonuclease for individual gRNA processing.
Zhang *et al.* [21]	Golden Gate assembly	8 gRNAs	tRNA processing	>2 days	No	gRNA spacer sequences being the Golden Gate overhangs restrict scalability.	2 (44–60 bp)	The gRNA maturation could interfere with endogenous tRNA pool.
Yuan *et al.* [22]	Golden Gate assembly	10 gRNAs	tRNA/Csy4/Ribozyme processing	3–5 days	No	gRNA spacer sequences being the Golden Gate overhangs restrict scalability.	2 (27–52 bp)	The gRNA maturation could interfere with endogenous tRNA pool (tRNA processing) or requires Csy4 endonuclease expression.
Breunig *et al.* [23]	Gibson assembly	8 gRNAs	Individual expression cassettes	3 days	No	gRNA spacer sequences mediate homology for Gibson assembly thus restricting scalability.	2 (36–45 bp)	Low assembly efficiency (34% for 4 gRNA assembly).

a Scalability indicates using the approach to assemble multiple gRNA arrays or libraries of arrays simultaneously*.*

**Figure 1:**
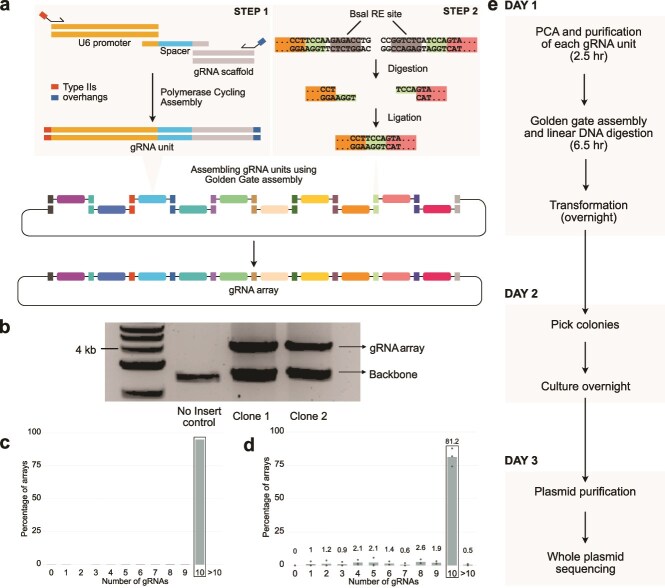
gRNA array assembly by RAPID-DASH. (a) Overview of RAPID-DASH. gRNA units are assembled using PCA with unique primer sets that add type IIS restriction enzyme recognition sites and 4 bp sequences that, when digested by BsaI, enable orderly assembly *via* Golden Gate assembly. The order of the assembly in this overview is determined by the colour-coded type IIS restriction site flanking the gRNA expression units. (b) Screening bacterial clones by digesting out the assembled arrays. Gel electrophoresis image shows bands at ~ 4 kb, which is the expected size for 10 gRNA arrays*.* (c and d) Bar plot showing the percentage of arrays with different numbers of gRNAs assembled from (c) a single-clone whole-plasmid picked *via* lacZ screening and (d) bulk plasmid sequencing from all the transformants, skipping the lacZ screening step, performed in triplicate. Highlighted bar shows 10 gRNA arrays. Numbers above bars indicate average values from the three replicates. (e) RAPID-DASH timeline.

A previous study [27] introduced a method to assemble multiple gRNAs into a single array using polymerase cycling assembly (PCA) [28] to generate the gRNA units, which are then assembled in a specific order and cloned into the vector using Golden Gate assembly [29] (Fig. 1). Using this approach, the authors assembled sets of five gRNAs into a single plasmid for multiplexed CRISPR-activation. Although this study demonstrated a modular way to assemble gRNA arrays, this method was not optimized or evaluated for assembly efficiency, error rates, or potential for scalable gRNA library generation. Building on this foundation, we optimized the approach into RAPID-DASH—**R**apid **A**ssembly of **P**CA-produced **I**ndividual **D**NAs and **D**irected **A**ssembly through type II**S** over**H**angs for assembling arrays of gRNAs that enables at least 10 gRNAs to be cloned into an array within a single day. Each gRNA unit is created by PCA of a dsDNA U6 promoter, an ssDNA gRNA spacer oligo, and dsDNA gRNA scaffold terminator, resulting in a single dsDNA gRNA unit. A unique primer set is used for each position of the ordered gRNA array, which, in addition to amplifying the assembled gRNA units, adds type IIS overhangs that enable ordered assembly of the gRNA units as an array (Fig. 1a, step 1). During the Golden Gate guide assembly process, BsaI, a type IIS restriction enzyme, digests the ends of each gRNA unit, exposing overhangs that are complementary only to the adjacent gRNA unit, facilitating correct orderly assembly by ligation (Fig. 1a step 2). The lacZ gene within the destination plasmid enables efficient identification of colonies with the gRNA array *via* blue-white colony screening [30] (Supplementary Fig. S1). In addition to cloning more gRNAs using RAPID-DASH, we provide a detailed characterization of the method, including measurement of assembly efficiency, quantitative error-rate profiling, and assessment of gRNA array library generation. These optimizations substantially enhance robustness, reduce synthesis costs, and establish RAPID-DASH as a versatile framework for constructing large, high-fidelity gRNA arrays suitable for multiplexed genome engineering applications.

## Methods

### gRNA unit generation using polymerase cycling assembly

Ten individual gRNA units were constructed by assembling double-stranded U6 promoter, single-stranded spacer sequence, and double-stranded terminator scaffold sequence into a single unit. The U6 promoter and gRNA terminator scaffold fragments were amplified from a commonly used gRNA expression vector (MLM3636). The spacer sequence was ordered from IDT as a single-stranded DNA oligo that included homology sequences to the U6 promoter and the terminator scaffold fragments. The assembly is mediated by a forward primer that binds to the U6 promoter and a reverse primer that binds to the terminator scaffold fragments. These primers were designed to include type II restriction enzyme (BsaI) overhangs that enable ordered assembly of the gRNA units into an array (Supplementary Material S1). We used the NEBridge Ligation fidelity GetSet tool to generate these type II overhangs (BsaI-HFv2 37-16 cycling). The sticky ends and the spacer oligos needed for assembling the gRNA units can be generated using our Shiny app: https://deboerlab.shinyapps.io/OligoDesigner/. The PCA reactions were set up as follows: 1 ul U6 promoter (5 ng), 1 ul gRNA terminator scaffold (5 ng), 2 ul spacer oligo (1 mM), 1.25 ul forward primers (10 uM), 1.25 ul reverse primers (10 uM), 25 ul Phusion High-Fidelity PCR Master Mix (Thermo F531), and nuclease-free water to a total volume of 50 ul. The following setting was used in thermocycler: 98°C for 3 min, 35 cycles of 98°C for 10 s, 52°C for 30 s, and 72°C for 12 s which was followed by a final 72°C for 8 min. The assembled units can be run on a 1% agarose gel to verify the length of units (433 bp). The gRNA units were then purified using AMPure XP beads. The PCA products were incubated with 1:1 ratio of AMPure XP beads and incubated for 5 min at room temperature. Following this, the product was placed on a magnetic separation rack and let the beads separate from the supernatant (~3 min). The supernatant was then removed from the tubes and the beads were rinsed with 80% ethanol twice. The beads were then air dried for maximum 5 min to remove any residual ethanol from washing. The tubes were taken off the magnetic rack and the beads were resuspended in 20 ul of warm nuclease-free water and incubated at room temperature for 5 min. The tubes were placed back on to the magnetic rack and the PCA products are eluted from the supernatant. This clean up step is critical for the high efficiency of Golden Gate assembly.

### gRNA array generation using Golden Gate assembly

The destination vector (pAL10) incorporating the Golden Gate cloning site into which the gRNA array is inserted was generated from pFUS-B10 vector from the TALEN assembly kit [31]. Golden Gate assembly for cloning gRNA arrays was set up as follows: 1 ul destination vector (pAL10) (100 ng), 1 ul each of the purified gRNA units (50 ng), 1 ul of T4 DNA ligase (2000 U/ul, NEB M0202T), 1.5 ul FastDigest Eco31I (Thermo FD0293) (BsaI isoschizomer), 2 ul of 10X T4 DNA ligase buffer (NEB), and nuclease-free water up to 20 ul. The following setting was used in the thermocycler: 5 min at 37°C and 5 min at 16°C for 30 cycles followed by 37°C for 10 min. The restriction enzyme was then inactivated at 75°C for 10 min. The reaction can be held at 4°C at this point. The reaction is mixed with 1 ul Plasmid-Safe ATP-Dependent DNase (Biosearch Technologies E3101K) and 1 ul of 25 mM ATP, and incubated at 37°C for 1 h. This step was crucial to avoid recombination of linear DNA after transformation in bacterial cells [18]. A total of 2 ul of the assembled product was used to transform NEB stable chemical competent cells using the manufacturer-supplied heat-shock transformation protocol and plated on LB agar with 50 ug/ml spectinomycin along with IPTG and X-gal for blue/white screening [30]. It is recommended to use recombination-deficient bacterial strains like NEB stable *Escherichia coli* to transform the assembled arrays to prevent recombination within array that could lead to gRNA dropout. The transformants were incubated at 30°C for 16 h. Plasmids isolated from white colonies were screened for correct gRNA array assembly *via* BsmBI-v2 digestion (NEB R0739, manufacturer’s protocol). For bulk plasmid sequencing, the transformants were directly inoculated into LB media with 50 ug/ml spectinomycin. Whole plasmid sequencing to validate the arrays was performed by Plasmidsaurus using Oxford Nanopore Technology.

The probability of picking a colony that is suitable for downstream use is estimated as follows. Mutations within the PCA primer regions are ignored in the following calculations as they are most likely to be inconsequential. All mutations indicated below only refer to mutations observed within either the U6 promoter, gRNA spacer or the gRNA scaffold of a gRNA unit.

The proportion of a gRNA units having a deleterious mutation is given by:


$$ p(M)= Number\ of\ gRNA\ units\ mutated/ Total\ number\ of\ gRNA\ units $$

Based on our data, *p*(*M*) = 7.5% (Supplementary Fig. S2).

The probability of a gRNA unit having no mutations is given by:


$$ p(G)=1-p(M) $$

Thus, *p*(*G*) = 92.5%.

The probability of an array with *k* gRNA units having no mutations, which assumes independence between gRNA units (they are synthesized separately) is given by:


$$ p(A)=p{(G)}^k $$

Our data suggest that the probability of an assembly being full length, $p(F)$, is 81% (Fig. 1d). This is assuming each of the nanopore reads from bulk plasmid sequencing stems from an individual clone and the assembly efficiency is the proportion of nanopore reads that are the full-length assembly.

The probability of a picked clone being a full-length assembly and having no mutations in any gRNA unit is given by:


$$ p(C)=p(A)p(F) $$

The probability of picking at least one usable clone (full-length assembly with no mutations) out of *n* colonies (assuming each is an independent transformation event) is given by:


$$ p(U)=1-{\left(1-p(C)\right)}^n $$

### GFP reporter cell line transfection

To validate the array, we used a mutated GFP reporter HEK293T cell line from Sakata *et al.* (2020) [32]. gRNA arrays consisting of the GFP-targeting gRNA within each position of the array were generated as above. We also included a gRNA array that did not include the GFP-targeting gRNA and plasmids that expressed individual gRNAs as controls. For the GFP reporter assay, 1 × 10^5^ cells were plated in wells of a 48-well plate the day before transfection. Cells were transfected with equimolar (40 fmoles) Target-AID base editor and either the single GFP-targeting gRNA plasmid or gRNA arrays in respective wells. Transporter5 reagent (Polysciences 26008) was used for transfecting the DNA into the cells using manufacturer’s protocol. Transfection Media was replaced with fresh media 24 h after transfection. Cells were harvested 72 h after transfection for flow cytometry analysis. Three independent replicates were performed for each transfection.

### Flow cytometry analysis

Cells were analysed for GFP expression using Cytoflex LX Analyser and gated using FlowJo (v10) (Supplementary Fig. S3). Singlet cells were extracted using FlowJo (v10) and then further processed using tidyverse (v2.0.0) in R 4.4.2.

### gRNA array library generation

To generate gRNA arrays with gRNAs randomly sampled from a pool, we pooled the 10 single-stranded spacer oligos in equimolar ratio prior to gRNA unit generation *via* PCA to mimic commercially synthesized oligo pools. gRNA units and the subsequent assembly were done as described above. Bulk plasmid sequencing was performed by Plasmidsaurus using Oxford Nanopore Technology.

### Nanopore sequencing analysis

The reads in fastq files from nanopore sequencing often contain the whole plasmid sequences. gRNA array sequences from the whole plasmid sequences were extracted using the ‘get_plasmid_inserts’ custom function which extracts the gRNA array inserts based on input flanking sequences in the backbone. This step discards any incomplete nanopore reads that lack the flanking sequences on the backbone and thus do not capture the whole plasmid. To gauge gRNA assembly efficiency, the extracted inserts were binned by length into the number of gRNA units each represents based on the known length of each gRNA unit (Supplementary Table S1). In order to estimate the mutation rates within the array clones, we aligned the consensus sequence from nanopore sequencing for each clone to the expected array sequence to identify any mutations within the gRNA units of the sequenced array (Supplementary Fig. S4). To get the distribution of gRNA sequences within the randomized gRNA array library, we filtered the nanopore reads to only full-length gRNA arrays with 10 gRNA units and used the RapidFuzz alignment [33] approach to map each gRNA sequence to the known sequences within the gRNA pool. The resultant gRNA versus position map was then plotted using ggplot2 (v3.5.1) in R 4.4.2. Detailed analysis can be found in our GitHub repository.

### Statistical analysis

Two-sample Welch’s *t*-test was performed on the GFP reporter flow cytometry data to compare the GFP reporter activation efficiency of each of the GFP-targeting guide in 10 guide positions to the non-targeting control and to the single GFP-targeting guide. Bonferroni’s method was used to adjust for multiple hypothesis testing within each comparison set (versus NTC and versus GFP-only). All the statistical analyses were performed in R 4.4.2. Detailed analysis can be found in our GitHub repository.

## Results and discussion

We tested RAPID-DASH to assemble an array of 10 gRNAs (see Methods section). Digestion of plasmids isolated from individual clones revealed that the assembled arrays are of expected size (4 kb) (Fig. 1b). To verify the sequence of the assembled gRNA arrays, we extracted plasmid from a single clone *via* lacZ screening and sequenced it using nanopore sequencing. We verified that it included all 10 gRNAs (Fig. 1c), and its gRNA sequences were correct (Supplementary Material S1) and assembled in the expected order. Whole plasmid sequencing of a pool of transformants (i.e. plasmids extracted from the recovered transformation mix grown in liquid LB + spectinomycin, without plating or lacZ screening) revealed that, on average, 81% (*n* = 3 replicates) of the assemblies have all 10 gRNA units (Fig. 1d; Supplementary Table S2). In order to estimate the error-rate within the assembly, we sequenced 28 gRNA array clones using nanopore sequencing. Error rates were estimated at the gRNA unit level, breaking it down into mutations that are presumably inconsequential as they are only used for gRNA unit amplification during PCA, and potentially deleterious mutations impacting the gRNA (U6 promoter, gRNA spacer, and scaffold), finding that 7.5% of the total 280 gRNA units had one or more potentially deleterious mutation (Supplementary Fig. S2). Given these results, screening of four colonies has an 84% chance of recovering a full-length array with no putatively deleterious mutations within any gRNA unit (see Methods section). Pre-screening the colonies by restriction digest can help eliminate incomplete assemblies (Fig. 1b). Furthermore, while we did not purify our oligos (standard desalting only), this would likely substantially reduce the error rate, although at a high cost per oligo. Depending on a laboratory’s individual circumstances, these may or may not be more economical than just directly screening colonies.

**Figure 2:**
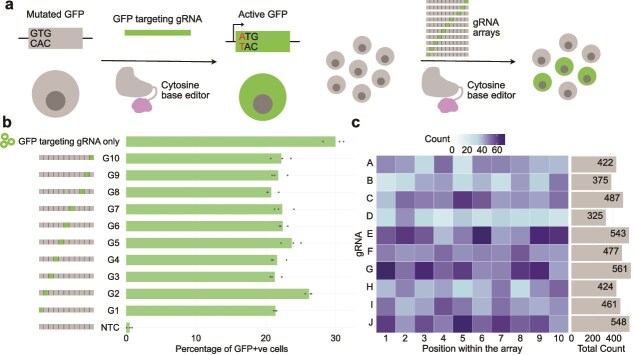
Functional validation of the assembled arrays. (a) GFP reporter assay for gRNA array validation. (b) Bar chart showing the percentage of the GFP reporter cells (x-axis) activated by gRNA arrays with GFP-targeting gRNA at each position (y-axis) within the array. NTC = non-targeting control with no GFP-targeting guide. (c) Abundance (colour) of each guide RNA (gRNA) sequences (y-axis) across different positions in an array (x-axis) within the nanopore reads of a bulk plasmid sequence when using pooled gRNA sequences during PCA for each array position. The bar plot on the right depicts the total count for each gRNA across all the 10 positions within the array.

To validate the functionality of gRNA units within the array, we used a cell line that can report successful CRISPR base editing by activating a GFP gene [32]. Here, the GFP gene is initially defective because its start codon has been mutated to GTG. By targeting the Target-AID base editor [34] to the start codon with a gRNA, the start codon is repaired to an ATG, enabling gRNA activity to be assayed easily *via* cells turning green (Fig. 2a). We cloned the GFP-targeting gRNA within each position of the gRNA arrays independently and observed that the 10 gRNA positions had similar efficacy, activating GFP in 20.17%–26.53% of cells (Fig. 2b), which was far more than the non-targeting gRNA control (0.48% GFP positive; Welch’s *t*-test *P* < .01 for all gRNAs; Supplementary Table S3). The efficacy of gRNAs in arrays was slightly less than that of a plasmid containing only a single GFP-targeting gRNA (30.56% GFP positive; .0218 ≤ *P* ≤ .419 for all gRNAs; Supplementary Table S3), which was expected due to the higher transfection efficiency of the smaller GFP-only construct and dilution effect caused by other GFP non-targeting guides competing with limited Cas9 to form ribonucleoproteins. These results illustrate that these gRNA arrays are suitable for multiplexed gene editing as all the positions within the array are functional and have similar editing efficiencies.

For applications requiring a randomized combinatorial genome editing or perturbation, it may be desirable to create libraries of gRNA arrays, each with gRNAs targeting distinct genomic loci. For example, in a large-scale CRISPRa/CRISPRi functional genomics screen, one might aim to interrogate the regulatory effect of ~ 100 000 genomic elements (e.g. promoters or enhancers) by randomly activating or repressing 10 elements per cell. This enables combinatorial perturbation, where different cells receive different combinations of targets, allowing higher-order interaction effects to be observed. By using pools of gRNA sequences during the PCA step, such libraries can be created with our approach. As a proof of concept, we tested this by including 10 gRNA sequences for each of the gRNA unit PCAs, and then assembled them as before into a library, where each library member gets an array of 10 gRNAs, each randomly sampled from the 10 possibilities. For most applications, one would want to sample from a larger pool of gRNAs, but for this demonstration we limited it to 10 so that we could obtain better sequencing coverage, although this strategy nonetheless could produce 10 billion (10^10^) unique correctly assembled arrays. Nanopore sequencing of these pooled arrays showed all 10 gRNAs were present in all 10 positions (Fig. 2c), albeit at a slightly reduced frequency of full-length assembly (69% versus 81.2%; Supplementary Fig. S5). Combined with the relatively low cost per oligo when ordering pooled oligos, this approach can be scaled up to include many more gRNA sequences to create libraries of gRNA arrays efficiently, enabling low-cost multiplexed perturbation assays. Of note, because the PCA of each gRNA unit is performed separately, one could limit the possible gRNAs in each position, ensuring that only certain combinations of gRNAs are created. For instance, this could be used to generate a gRNA array where each position targets a subset of genes, with different subsets targeted in each gRNA position, ensuring every array will target exactly one gene within each subset.

Using RAPID-DASH, we can generate and validate gRNA arrays with only a single day of hands-on time (Fig. 1e). RAPID-DASH offers substantial cost savings because only a short (~60 nt) spacer sequence needs to be ordered for each new gRNA. Researchers can also skip the PCA step if they have gRNA units synthesized (433 bp; e.g. gBlocks); however, this will increase the cost substantially as the PCA-based synthesis requires only a single 59 nt spacer oligo for each new gRNA unit. Furthermore, the high-efficiency of our approach saves additional time and labour, reducing the need for layers of screening and enabling one to use libraries of gRNA arrays directly in downstream applications. The major bottlenecks of RAPID-DASH reflect oligo synthesis and sequencing.

Although we only scale to 10 gRNA units, which should satisfy most applications for the time being, we anticipate that RAPID-DASH can be scaled further by using appropriate type II restriction enzyme overhangs to assemble up to 52 gRNA units, although this would result in a plasmid of substantial size (~24 kb) and likely reduce the efficiency of the assembly and stability of the assembled arrays. RAPID-DASH enables faster and more efficient construction and more robust delivery of gRNA arrays, facilitating rapid experimental iteration at scale (Table 1). In order to facilitate adoption of RAPID-DASH, we developed a Shiny app (https://deboerlab.shinyapps.io/OligoDesigner/) that enables design of spacer sequence oligos for assembly of up to 20 gRNA units within a single array. Arrays assembled using RAPID-DASH can be transfected directly, used to create virus-like particles containing CRISPR ribonucleoproteins [35], or cloned into PiggyBac vectors furthering the scale to perform multiplexed CRISPR screens [36]. Although lentivirus is commonly used for CRISPR screens, long and repetitive gRNA arrays, such as those assembled using RAPID-DASH are poorly compatible with lentiviral delivery as they reduce viral titres and undergo reverse-transcription–mediated recombination that leads to gRNA dropout within the array. Readouts of CRISPR screens vary by experiment, but we anticipate that RAPID-DASH will be most useful for approaches that read out the mutations directly [37], incorporate a barcode that can be used to infer the guide array [38], or *via* directly capturing and sequencing gRNAs (10X CRISPR Guide Capture) [17], where imputation could be used to infer gRNA presence even when unobserved if it was known to be on the same array.

## Supplementary Material

Supplementary_Material-Complete_ysaf020

## Data Availability

Raw nanopore sequencing of assembled arrays are available at https://github.com/de-Boer-Lab/RAPID-DASH/. Code used for processing nanopore data and plotting are available at the https://github.com/de-Boer-Lab/RAPID-DASH/

## Supplementary data

The supplementary materials (.docx) file contains blue-white screening of bacterial colonies following gRNA array assembly (Supplementary Fig. S1), percentage of gRNA units mutated within the screened arrays (Supplementary Fig. S2), gating summary for flow cytometry data (Supplementary Fig. S3), example mutations seen in gRNA arrays (Supplementary Fig. S4), bar plot showing the percentage of arrays with different numbers of gRNAs assembled within the combinatorial gRNA array library (Supplementary Fig. S5), read length binning thresholds for calculating the number of gRNAs in an array (Supplementary Table S1), gRNA array assembly efficiency as a percentage from bulk plasmid sequencing (Supplementary Table S2), pairwise comparisons of GFP reporter activation for each gRNA position within the array *versus* NTC and GFP-only controls (Supplementary Table S3) and the oligos and sequences for gRNA assembly. Supplementary material also contain the alignment of a full-length clonal gRNA array plasmid sequence generated *via* nanopore sequencing to the desired sequence.

## Material availability

Destination vector plasmid (pAL10) used to clone the gRNA arrays is deposited to Addgene for non-commercial use (Addgene #236006).
