# Supplementary material for: RAPID-DASH: fast and efficient assembly of guide RNA arrays for multiplexed CRISPR-Cas9 applications
Source: Synth Biol (Oxf). 2025 Dec 18;11(1):ysaf020. doi: 10.1093/synbio/ysaf020 (PMC12828702; doi:10.1093/synbio/ysaf020)
Supplement: Supplementary_Material-Complete_ysaf020 [file supplementary_material-complete_ysaf020.pdf]

RAPID-DASH: Single-Day Assembly of Guide RNA Arrays for Multiplexed CRISPR-Cas9 Applications

Asfar Lathif Salaudeen[1], Nicholas Mateyko[1], Carl G. de Boer[2*]

[1]Genome Science and Technology Graduate Program, University of British Columbia, Vancouver, BC, Canada[2]School of Biomedical Engineering, University of British Columbia, Vancouver, BC, Canada

*Corresponding Author – carl.deboer@ubc.ca

Supplementary Materials

This document includes the following:

Figure S1

Figure S2

Figure S3

Figure S4

Figure S5

Table S1

Table S2

Table S3

Oligos and sequences for gRNA assembly

Consensus sequence of a clonal 10 gRNA assembly

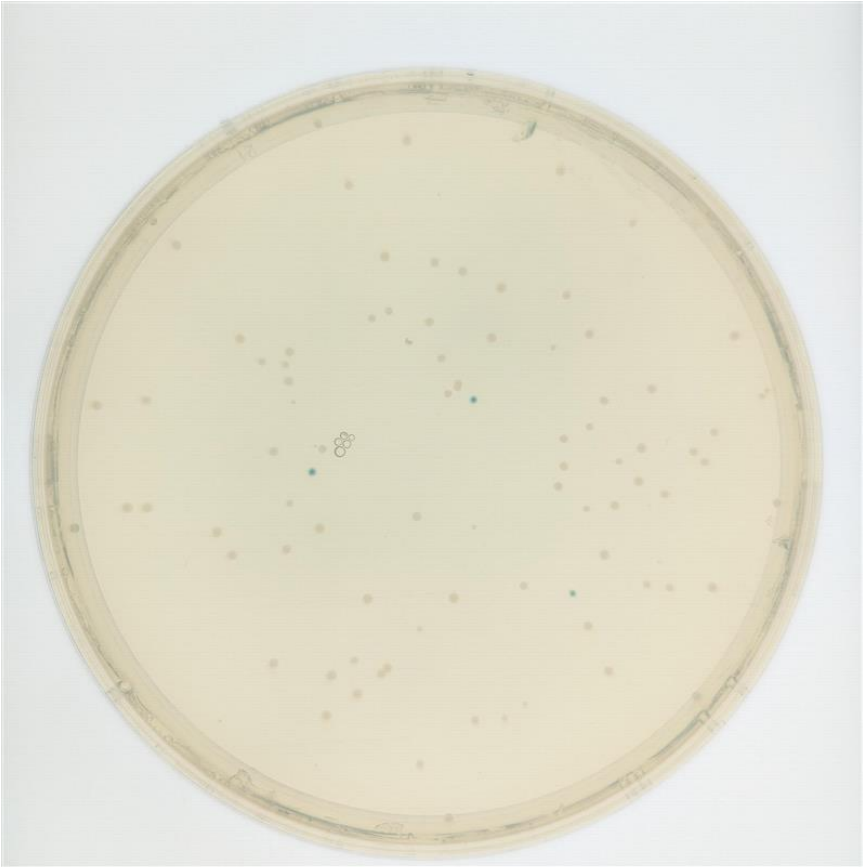

Figure S1: Example blue-white screening of bacterial colonies following gRNA array assembly. White colonies indicate that there was an insertion into the gRNA array cloning site, while blue colonies represent vectors with no insertion.

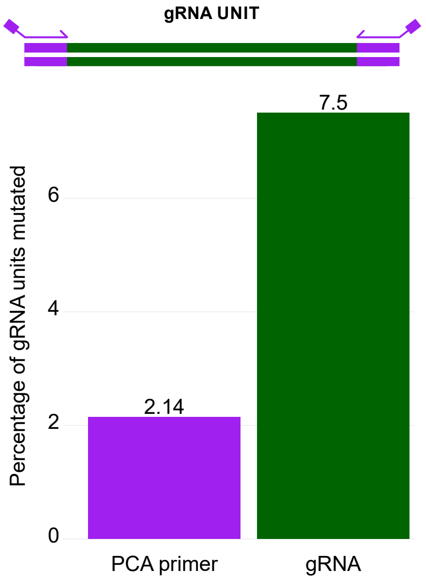

Figure S2: Percentage of gRNA units mutated within the screened arrays. Mutations observed within the gRNA units in 28 gRNA array clones were categorized based on their

occurrence in the PCA primer binding region or the rest of the gRNA units, including the U6 promoter, spacer sequence, and the gRNA scaffold.

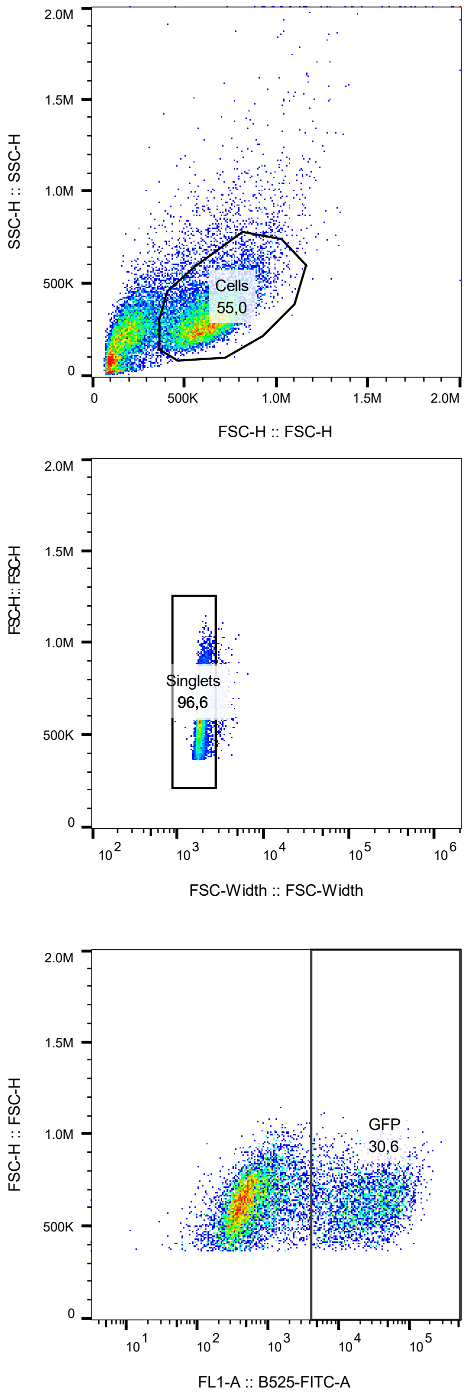

Figure S3: Gating summary for flow cytometry data for GFP reporter activation experiments. Cells were gated for singlets and then GFP positive cells using FlowJo (v10). Forward vs side scatter height plot was used to gate cells from cellular debris (top), which was

then gated for singlets using forward scatter width vs height (middle) and FITC-A channel was used to gate for GFP positive cells from the singlets (bottom).

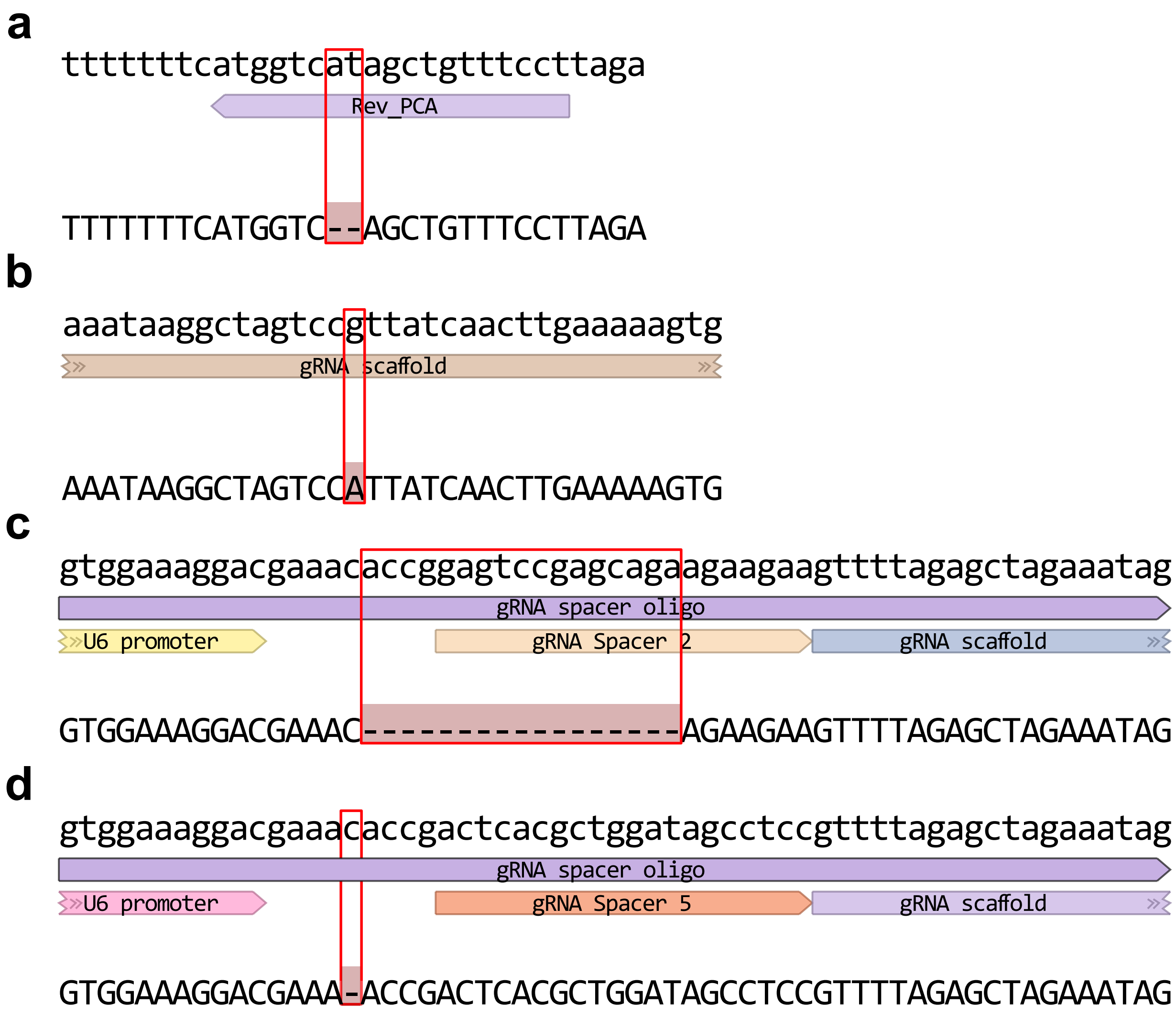

Figure S4: Example mutations seen in gRNA arrays. Alignments of the consensus sequence of the screened gRNA array clones (bottoms) with desired gRNA assembly plasmid sequence (tops). a) two bp deletion in the priming part of a PCA handle primer. b) 1 bp substitution in the priming part of a PCR handle primer. c) 17 bp deletion and (d) a 1 bp deletion within gRNA spacer oligo

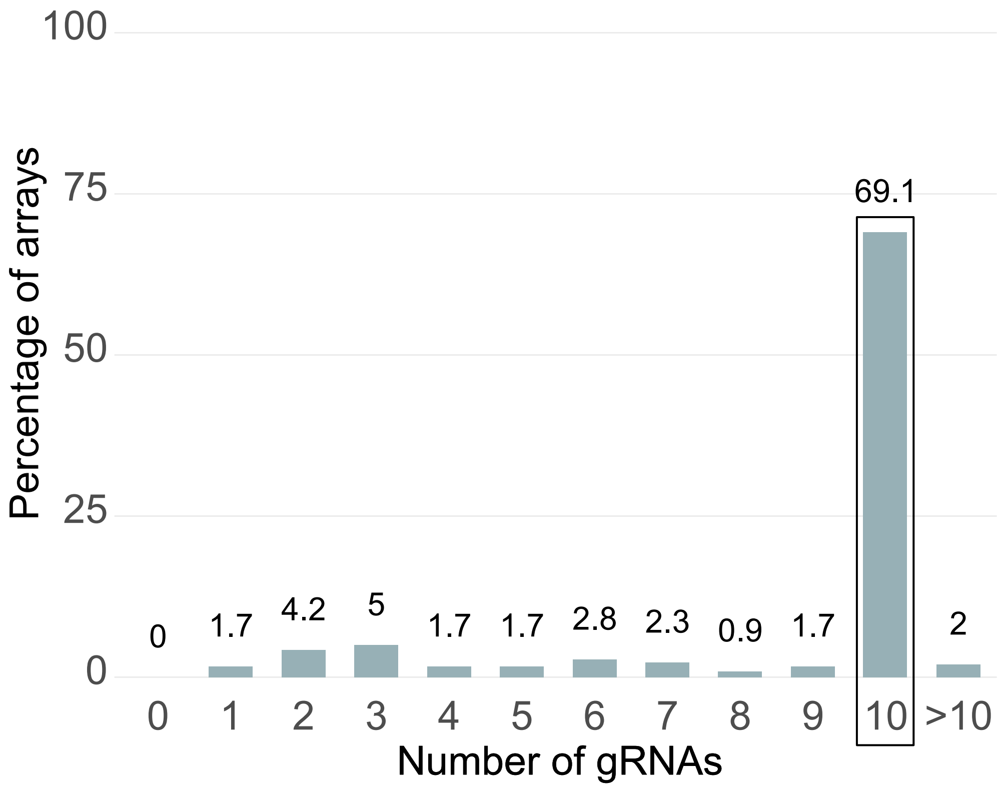

Figure S5: Bar plot showing the percentage of arrays with different number of gRNAs assembled within the combinatorial gRNA array library. Highlighted bar shows full-length 10 gRNA arrays. Efficiency is likely decreased due to the even more repetitive nature of these arrays (only 10 possible spacers were cloned and into every position, so the vast majority have verbatim duplicated gRNA units), potentially leading to increased recombination and gRNA unit loss.

Table S1: Read length binning thresholds for calculating the number of gRNAs in an array. Expected insert lengths were calculated based on the number of gRNAs in an array. Ranges were used to account for high error rate of nanopore sequencing.

Number of gRNAs	Expected Length (bp)	Lower range (bp)	Upper Range (bp)
>10	>4311	4241	
10	3917	3847	3987
9	3523	3453	3593
8	3129	3059	3199
7	2735	2665	2805
6	2341	2271	2411
5	1947	1877	2017
4	1553	1483	1623
3	1159	1089	1229
2	765	695	835
1	371	301	441

0	0	0	70

Table S2: gRNA array assembly. Values represent the percentages of extracted gRNA array inserts (from bulk whole plasmid nanopore reads) whose lengths correspond to each number of gRNA units in the array. Full-length 10-gRNA arrays is highlighted in green. "Unclassified" represents inserts that did not match expected array sizes.

Number of gRNAs in the array	Assembly 1	Assembly 2	Assembly 3
**0**	0	0	0
**1**	3.03	0	0
**2**	0	0	3.68
**3**	0	0	2.63
**4**	1.52	0.58	4.21
**5**	0	0.58	5.79
**6**	3.03	0.58	0.53
**7**	0	1.75	0
**8**	4.55	1.75	1.58
**9**	1.52	1.17	3.16
**10**	81.82	87.72	74.21
**>10**	0	0.58	1.05
**Unclassified**	4.55	5.26	3.16

Table S3: Pairwise comparisons of GFP reporter activation for each gRNA position within the array versus non-targeting controls (NTC) and GFP-only controls. p-values were calculated using two-sample t-tests and adjusted for multiple comparisons using the Bonferroni method within each comparison set (vs. NTC and vs. GFP-only).

Comparison	p-values	Adjusted p-values	Significance
G1 vs NTC	3.66e-06	3.66e-05	***
G2 vs NTC	4.16e-07	4.16e-06	***
G3 vs NTC	8.91e-05	8.91e-04	***
G4 vs NTC	3.67e-04	3.67e-03	**
G5 vs NTC	6.47e-04	6.47e-03	**
G6 vs NTC	6.27e-06	6.27e-05	***
G7 vs NTC	8.11e-04	8.11e-03	**
G8 vs NTC	1.24e-04	1.24e-03	**
G9 vs NTC	3.64e-04	3.64e-03	**
G10 vs NTC	7.72e-04	7.72e-03	**
G1 vs GFP only	9.74e-03	9.74e-02	
G2 vs GFP only	4.19e-02	4.19e-01	
G3 vs GFP only	2.78e-03	2.78e-02	*
G4 vs GFP only	2.29e-03	2.29e-02	*
G5 vs GFP only	7.01e-03	7.01e-02	
G6 vs GFP only	6.72e-03	6.72e-02	
G7 vs GFP only	3.57e-03	3.57e-02	*
G8 vs GFP only	2.18e-03	2.18e-02	*
G9 vs GFP only	2.45e-03	2.45e-02	*
G10 vs GFP only	3.26e-03	3.26e-02	*

Statistical significance is marked with * as follows: * - adjusted p-value < 0.05, ** - adjusted p-value < 0.01 and *** - adjusted p-value < 0.001.

Oligos and sequences for gRNA assembly

Note: Based on our finding that the primers are a primary cause of mutations in final gRNA array assemblies, the forward and reverse primers for the initial amplification of the U6 promoter and gRNA terminator scaffold are one of the only places that we think makes economic sense to purify (e.g. HPLC to increase the purity of full-length oligos). This is because the PCR products they produce are used in every single gRNA unit and they could introduce mutations that adversely affect gRNA function. The gRNA unit amplification oligos are priming in inconsequential regions and so any introduced mutations are likely benign, and gRNA spacer oligos are often used only once and so it may or may not be economical to purify them. The results in this study reflect no purification of any oligos.

U6 Promoter

Forward primer: GTAAAACGACGGCCAGTgagggcctatttcccatgattc

Reverse primer: GGTGTTTCGTCCTTTCCAC

Amplicon sequence:
GTAAAACGACGGCCAGTgagggcctatttcccatgattccttcatatttgcatatacgatacaaggctgttagagagataattggaattaatttgactgtaaacacaaagatattagtacaaaatacgtgacgtagaaagtaataatttcttgggtagtttgcagttttaaaattatgttttaaaatggactatcatatgcttaccgtaacttgaaagtatttcgatttcttggctttatatatcttGTGGAAAGGACGAAACACC

gRNA terminator scaffold

Forward primer: gttttagagctaGAAAtagcaag

Reverse primer: AGGAAACAGCTATGACCATGAAAAAAAgcaccgactcggtgccac

Amplicon sequence:
gttttagagctaGAAAtagcaagttaaaataaggctagtccgttatcaacttgaaaaagtggcaccgagtcggtgcTTTTTTTCATGGTCATAGCTGTTTCCT

gRNA spacer oligos

Sample:
GTGGAAAGGACGAAACACCgNNNNNNNNNNNNNNNNNNNNgttttagagctaGAAAtag

	gRNA spacer	Ordered oligo
1	GGAATCCCTTCTGCAGCACC	GTGGAAAGGACGAAACACCgGGAATCCCTTCTGCAGCACCgttttagagctaGAAAtag
2	GAGTCCGAGCAGAAGAAGAA	GTGGAAAGGACGAAACACCgGAGTCCGAGCAGAAGAAGAAgttttagagctaGAAAtag
3	TTTATCACAGGCTCCAGGAA	GTGGAAAGGACGAAACACCgTTTATCACAGGCTCCAGGAAgttttagagctaGAAAtag
4	GGCCCAGACTGAGCACGTGA	GTGGAAAGGACGAAACACCgGGCCCAGACTGAGCACGTGAgttttagagctaGAAAtag
5	ACTCACGCTGGATAGCCTCC	GTGGAAAGGACGAAACACCgACTCACGCTGGATAGCCTCCgttttagagctaGAAAtag
6	GTCATCTTAGTCATTACCTG	GTGGAAAGGACGAAACACCgGTCATCTTAGTCATTACCTGgttttagagctaGAAAtag
7	GGCACTGCGGCTGGAGGTGG	GTGGAAAGGACGAAACACCgGGCACTGCGGCTGGAGGTGGgttttagagctaGAAAtag
8	CACCTACCTAAGAACCATCC	GTGGAAAGGACGAAACACCgCACCTACCTAAGAACCATCCgttttagagctaGAAAtag
9	TTCGTATCTGTAAAACCAAG	GTGGAAAGGACGAAACACCgTTCGTATCTGTAAAACCAAGgttttagagctaGAAAtag
10	GTATCTAGTGTTGGTGTCCT	GTGGAAAGGACGAAACACCgGTATCTAGTGTTGGTGTCCTgttttagagctaGAAAtag
GFP targeting	CACGGTCACCCTGACACGCT	GTGGAAAGGACGAAACACCgCACGGTCACCCTGACACGCTgttttagagctaGAAAtag

Primers for gRNA unit amplification

gRNA unit	Primer direction	Sequence	BsaI Overhang
1	Fwd	ATAAGGATCCGGTCTCAGGTAGTAAAACGACGGCCAGT	GGTA
1	Rev	ATAATGTACAGGTCTCTTCTAAGGAAACAGCTATGACCATG	TCTA
2	Fwd	ATAAGGATCCGGTCTCATAGAGTAAAACGACGGCCAGT	TAGA
2	Rev	ATAATGTACAGGTCTCTGGAGAGGAAACAGCTATGACCATG	GGAG
3	Fwd	ATAAGGATCCGGTCTCACTCCGTAAAACGACGGCCAGT	CTCC
3	Rev	ATAATGTACAGGTCTCTTGATAGGAAACAGCTATGACCATG	TGAT
4	Fwd	ATAAGGATCCGGTCTCAATCAGTAAAACGACGGCCAGT	ATCA
4	Rev	ATAATGTACAGGTCTCTTCAGAGGAAACAGCTATGACCATG	TCAG
5	Fwd	ATAAGGATCCGGTCTCACTGAGTAAAACGACGGCCAGT	CTGA
5	Rev	ATAATGTACAGGTCTCTCGCTAGGAAACAGCTATGACCATG	CGCT
6	Fwd	ATAAGGATCCGGTCTCAAGCGGTAAAACGACGGCCAGT	AGCG
6	Rev	ATAATGTACAGGTCTCTCCTTAGGAAACAGCTATGACCATG	CCTT
7	Fwd	ATAAGGATCCGGTCTCAAAGGGTAAAACGACGGCCAGT	AAGG
7	Rev	ATAATGTACAGGTCTCTGATGAGGAAACAGCTATGACCATG	GATG
8	Fwd	ATAAGGATCCGGTCTCACATCGTAAAACGACGGCCAGT	CATC
8	Rev	ATAATGTACAGGTCTCTAGGTAGGAAACAGCTATGACCATG	AGGT
9	Fwd	ATAAGGATCCGGTCTCAACCTGTAAAACGACGGCCAGT	ACCT
9	Rev	ATAATGTACAGGTCTCTTCGCAGGAAACAGCTATGACCATG	TCGC
10	Fwd	ATAAGGATCCGGTCTCAGCGAGTAAAACGACGGCCAGT	GCGA
10	Rev	ATAATGTACAGGTCTCTTACGAGGAAACAGCTATGACCATG	TACG

Consensus sequence of a clonal 10 gRNA assembly:

```
             1                                                                                 82
Template     ttgagatcctttttttctgcgcgtaatctgctgcttgcaaacaaaaaaaccaccgctaccagcggtggtttgtttgccggat
Clonal Se... TTGAGATCCTTTTTTTCTGCGCGTAATCTGCTGCTTGCAAACAAAAAAACCACCGCTACCAGCGGTGGTTTGTTTGCCGGAT

.............................................................................................

             83                                                                               164
Template     caagagctaccaactctttttccgaaggtaactggcttcagcagagcgcagataccaaatactgttcttctagtgtagccgt
Clonal Se... CAAGAGCTACCAACTCTTTTTCCGAAGGTAACTGGCTTCAGCAGAGCGCAGATACCAAATACTGTTCTTCTAGTGTAGCCGT

.............................................................................................

             165                                                                              246
Template     agttaggccaccacttcaagaactctgtagcaccgcctacatacctcgctctgctaatcctgttaccagtggctgctgccag
Clonal Se... AGTTAGGCCACCACTTCAAGAACTCTGTAGCACCGCCTACATACCTCGCTCTGCTAATCCTGTTACCAGTGGCTGCTGCCAG

.............................................................................................

             247                                                                              328
Template     tggcgataagtcgtgtcttaccgggttggactcaagacgatagttaccggataaggcgcagcggtcgggctgaacggggggt
Clonal Se... TGGCGATAAGTCGTGTCTTACCGGGTTGGACTCAAGACGATAGTTACCGGATAAGGCGCAGCGGTCGGGCTGAACGGGGGGT

.............................................................................................

             329                                                                              410
Template     tcgtgcacacagcccagcttggagcgaacgacctacaccgaactgagatacctacagcgtgagctatgagaaagcgccacgc
Clonal Se... TCGTGCACACAGCCCAGCTTGGAGCGAACGACCTACACCGAACTGAGATACCTACAGCGTGAGCTATGAGAAAGCGCCACGC

.............................................................................................

             411                                                                              492
Template     ttcccgaagggagaaaggcggacaggtatccggtaagcggcagggtcggaacaggagagcgcacgagggagcttccaggggg
Clonal Se... TTCCCGAAGGGAGAAAGGCGGACAGGTATCCGGTAAGCGGCAGGGTCGGAACAGGAGAGCGCACGAGGGAGCTTCCAGGGGG

.............................................................................................

             493                                                                              574
Template     aaacgcctggtatctttatagtcctgtcgggtttcgccacctctgacttgagcgtcgatttttgtgatgctcgtcagggggg
Clonal Se... AAACGCCTGGTATCTTTATAGTCCTGTCGGGTTTCGCCACCTCTGACTTGAGCGTCGATTTTTGTGATGCTCGTCAGGGGGG

.............................................................................................

             575                                                                              656
Template     cggagcctatggaaaaacgccagcaacgcggcctttttacggttcctggccttttgctggccttttgctcacatgttctttc
Clonal Se... CGGAGCCTATGGAAAAACGCCAGCAACGCGGCCTTTTTACGGTTCCTGGCCTTTTGCTGGCCTTTTGCTCACATGTTCTTTC

.............................................................................................

             657                                                                              738
Template     ctgcgttatcccctgattctgtggataaccgtattaccgcctttgagtgagctgataccgctcgccgcagccgaacgaccga
Clonal Se... CTGCGTTATCCCCTGATTCTGTGGATAACCGTATTACCGCCTTTGAGTGAGCTGATACCGCTCGCCGCAGCCGAACGACCGA

.............................................................................................

             739                                                                              820
Template     gcgcagcgagtcagtgagcgaggaagcggaagagcgcccaatacgcaaaccgcctctccccgcgcgttggccgattcattaa
Clonal Se... GCGCAGCGAGTCAGTGAGCGAGGAAGCGGAAGAGCGCCCAATACGCAAACCGCCTCTCCCCGCGCGTTGGCCGATTCATTAA

.............................................................................................

             821                                                                              902
Template     tgcagctggcacgacaggtttcccgactggaaagcgggcagtgagcgcaacgcaattaatacgcgtaccgctagccaggaag
Clonal Se... TGCAGCTGGCACGACAGGTTTCCCGACTGGAAAGCGGGCAGTGAGCGCAACGCAATTAATACGCGTACCGCTAGCCAGGAAG

.............................................................................................
```

```
             903                                                                            984
Template     agtttgtagaaacgcaaaaaggccatccgtcaggatggccttctgcttagtttgatgcctggcagtttatggcgggcgtcct
Clonal Se... AGTTTGTAGAAACGCAAAAAGGCCATCCGTCAGGATGGCCTTCTGCTTAGTTTGATGCCTGGCAGTTTATGGCGGGCGTCCT

...............................................................................................

             985                                                                           1066
Template     gcccgccaccctccgggccgttgcttcacaacgttcaaatccgctcccggcggatttgtcctactcaggagagcgttcaccg
Clonal Se... GCCCGCCACCCTCCGGGCCGTTGCTTCACAACGTTCAAATCCGCTCCCGGCGGATTTGTCCTACTCAGGAGAGCGTTCACCG

...............................................................................................

             1067                                                                          1148
Template     acaaacaacagataaaacgaaaggcccagtcttccgactgagcctttcgttttatttgatgcctggcagttccctactctcg
Clonal Se... ACAAACAACAGATAAAACGAAAGGCCCAGTCTTCCGACTGAGCCTTTCGTTTTATTTGATGCCTGGCAGTTCCCTACTCTCG

...............................................................................................

             1149                                                                          1230
Template     cgttaacgctagcatggatgttttcccagtcacgacgttgtaaaacgacggccagtcttaagcgtctcatggcctgaccccg
Clonal Se... CGTTAACGCTAGCATGGATGTTTTCCCAGTCACGACGTTGTAAAACGACGGCCAGTCTTAAGCGTCTCATGGCCTGACCCCG

...............................................................................................

             1231                                                                          1312
Template     gaccaagtggtggggtagtaaaacgacggccagtgagggcctatttcccatgattccttcatatttgcatatacgatacaag
Clonal Se... GACCAAGTGGTGGGGTAGTAAAACGACGGCCAGTGAGGGCCTATTTCCCATGATTCCTTCATATTTGCATATACGATACAAG

...............................................................................................

             1313                                                                          1394
Template     gctgttagagagataattagaattaatttgactgtaaacacaaagatattagtacaaaatacgtgacgtagaaagtaataat
Clonal Se... GCTGTTAGAGAGATAATTAGAATTAATTTGACTGTAAACACAAAGATATTAGTACAAAATACGTGACGTAGAAAGTAATAAT

...............................................................................................

             1395                                                                          1476
Template     ttcttgggtagtttgcagttttaaaattatgttttaaaatggactatcatatgcttaccgtaacttgaaagtatttcgattt
Clonal Se... TTCTTGGGTAGTTTGCAGTTTTAAAATTATGTTTTAAAATGGACTATCATATGCTTACCGTAACTTGAAAGTATTTCGATTT

...............................................................................................

             1477                                                                          1558
Template     cttggctttatatatcttgtggaaaggacgaaacaccgggaatcccttctgcagcaccgttttagagctagaaatagcaagt
Clonal Se... CTTGGCTTTATATATCTTGTGGAAAGGACGAAACACCGGGAATCCCTTCTGCAGCACCGTTTTAGAGCTAGAAATAGCAAGT

...............................................................................................

             1559                                                                          1640
Template     taaaataaggctagtccgttatcaacttgaaaaagtggcaccgagtcggtgctttttttcatggtcatagctgtttccttag
Clonal Se... TAAAATAAGGCTAGTCCGTTATCAACTTGAAAAAGTGGCACCGAGTCGGTGCTTTTTTTCATGGTCATAGCTGTTTCCTTAG

...............................................................................................

             1641                                                                          1722
Template     agtaaaacgacggccagtgagggcctatttcccatgattccttcatatttgcatatacgatacaaggctgttagagagataa
Clonal Se... AGTAAAACGACGGCCAGTGAGGGCCTATTTCCCATGATTCCTTCATATTTGCATATACGATACAAGGCTGTTAGAGAGATAA

...............................................................................................

             1723                                                                          1804
Template     ttagaattaatttgactgtaaacacaaagatattagtacaaaatacgtgacgtagaaagtaataatttcttgggtagtttgc
Clonal Se... TTAGAATTAATTTGACTGTAAACACAAAGATATTAGTACAAAATACGTGACGTAGAAAGTAATAATTTCTTGGGTAGTTTGC

...............................................................................................
```

```
              1805                                                                            1886
Template      agttttaaaattatgttttaaaatggactatcatatgcttaccgtaacttgaaagtatttcgatttcttggctttatatatc
Clonal Se...  AGTTTTAAAATTATGTTTTAAAATGGACTATCATATGCTTACCGTAACTTGAAAGTATTTCGATTTCTTGGCTTTATATATC

..................................................................................................

              1887                                                                            1968
Template      ttgtggaaaggacgaaacaccggagtccgagcagaagaagaagttttagagctagaaatagcaagttaaaataaggctagtc
Clonal Se...  TTGTGGAAAGGACGAAACACCGGAGTCCGAGCAGAAGAAGAAGTTTTAGAGCTAGAAATAGCAAGTTAAAATAAGGCTAGTC

..................................................................................................

              1969                                                                            2050
Template      cgttatcaacttgaaaaagtggcaccgagtcggtgctttttttcatggtcatagctgtttcctctccgtaaaacgacggcca
Clonal Se...  CGTTATCAACTTGAAAAAGTGGCACCGAGTCGGTGCTTTTTTTCATGGTCATAGCTGTTTCCTCTCCGTAAAACGACGGCCA

..................................................................................................

              2051                                                                            2132
Template      gtgagggcctatttcccatgattccttcatatttgcatatacgatacaaggctgttagagagataattagaattaatttgac
Clonal Se...  GTGAGGGCCTATTTCCCATGATTCCTTCATATTTGCATATACGATACAAGGCTGTTAGAGAGATAATTAGAATTAATTTGAC

..................................................................................................

              2133                                                                            2214
Template      tgtaaacacaaagatattagtacaaaatacgtgacgtagaaagtaataatttcttgggtagtttgcagttttaaaattatgt
Clonal Se...  TGTAAACACAAAGATATTAGTACAAAATACGTGACGTAGAAAGTAATAATTTCTTGGGTAGTTTGCAGTTTTAAAATTATGT

..................................................................................................

              2215                                                                            2296
Template      tttaaaatggactatcatatgcttaccgtaacttgaaagtatttcgatttcttggctttatatatcttgtggaaaggacgaa
Clonal Se...  TTTAAAATGGACTATCATATGCTTACCGTAACTTGAAAGTATTTCGATTTCTTGGCTTTATATATCTTGTGGAAAGGACGAA

..................................................................................................

              2297                                                                            2378
Template      acaccgtttatcacaggctccaggaagttttagagctagaaatagcaagttaaaataaggctagtccgttatcaacttgaaa
Clonal Se...  ACACCGTTTATCACAGGCTCCAGGAAGTTTTAGAGCTAGAAATAGCAAGTTAAAATAAGGCTAGTCCGTTATCAACTTGAAA

..................................................................................................

              2379                                                                            2460
Template      aagtggcaccgagtcggtgctttttttcatggtcatagctgtttcctatcagtaaaacgacggccagtgagggcctatttcc
Clonal Se...  AAGTGGCACCGAGTCGGTGCTTTTTTTCATGGTCATAGCTGTTTCCTATCAGTAAAACGACGGCCAGTGAGGGCCTATTTCC

..................................................................................................

              2461                                                                            2542
Template      catgattccttcatatttgcatatacgatacaaggctgttagagagataattagaattaatttgactgtaaacacaaagata
Clonal Se...  CATGATTCCTTCATATTTGCATATACGATACAAGGCTGTTAGAGAGATAATTAGAATTAATTTGACTGTAAACACAAAGATA

..................................................................................................

              2543                                                                            2624
Template      ttagtacaaaatacgtgacgtagaaagtaataatttcttgggtagtttgcagttttaaaattatgttttaaaatggactatc
Clonal Se...  TTAGTACAAAATACGTGACGTAGAAAGTAATAATTTCTTGGGTAGTTTGCAGTTTTAAAATTATGTTTTAAAATGGACTATC

..................................................................................................

              2625                                                                            2706
Template      atatgcttaccgtaacttgaaagtatttcgatttcttggctttatatatcttgtggaaaggacgaaacaccgggcccagact
Clonal Se...  ATATGCTTACCGTAACTTGAAAGTATTTCGATTTCTTGGCTTTATATATCTTGTGGAAAGGACGAAACACCGGGCCCAGACT

..................................................................................................
```

```
             2707                                                                           2788
Template     gagcacgtgagttttagagctagaaatagcaagttaaaataaggctagtccgttatcaacttgaaaaagtggcaccgagtcg
Clonal Se... GAGCACGTGAGTTTTAGAGCTAGAAATAGCAAGTTAAAATAAGGCTAGTCCGTTATCAACTTGAAAAAGTGGCACCGAGTCG

...............................................................................................

             2789                                                                           2870
Template     gtgctttttttcatggtcatagctgtttcctctgagtaaaacgacggccagtgagggcctatttcccatgattccttcatat
Clonal Se... GTGCTTTTTTTCATGGTCATAGCTGTTTCCTCTGAGTAAAACGACGGCCAGTGAGGGCCTATTTCCCATGATTCCTTCATAT

...............................................................................................

             2871                                                                           2952
Template     ttgcatatacgatacaaggctgttagagagataattagaattaatttgactgtaaacacaaagatattagtacaaaatacgt
Clonal Se... TTGCATATACGATACAAGGCTGTTAGAGAGATAATTAGAATTAATTTGACTGTAAACACAAAGATATTAGTACAAAATACGT

...............................................................................................

             2953                                                                           3034
Template     gacgtagaaagtaataatttcttgggtagtttgcagttttaaaattatgttttaaaatggactatcatatgcttaccgtaac
Clonal Se... GACGTAGAAAGTAATAATTTCTTGGGTAGTTTGCAGTTTTAAAATTATGTTTTAAAATGGACTATCATATGCTTACCGTAAC

...............................................................................................

             3035                                                                           3116
Template     ttgaaagtatttcgatttcttggctttatatatcttgtggaaaggacgaaacaccgactcacgctggatagcctccgtttta
Clonal Se... TTGAAAGTATTTCGATTTCTTGGCTTTATATATCTTGTGGAAAGGACGAAACACCGACTCACGCTGGATAGCCTCCGTTTTA

...............................................................................................

             3117                                                                           3198
Template     gagctagaaatagcaagttaaaataaggctagtccgttatcaacttgaaaaagtggcaccgagtcggtgctttttttcatgg
Clonal Se... GAGCTAGAAATAGCAAGTTAAAATAAGGCTAGTCCGTTATCAACTTGAAAAAGTGGCACCGAGTCGGTGCTTTTTTTCATGG

...............................................................................................

             3199                                                                           3280
Template     tcatagctgtttcctagcggtaaaacgacggccagtgagggcctatttcccatgattccttcatatttgcatatacgataca
Clonal Se... TCATAGCTGTTTCCTAGCGGTAAAACGACGGCCAGTGAGGGCCTATTTCCCATGATTCCTTCATATTTGCATATACGATACA

...............................................................................................

             3281                                                                           3362
Template     aggctgttagagagataattagaattaatttgactgtaaacacaaagatattagtacaaaatacgtgacgtagaaagtaata
Clonal Se... AGGCTGTTAGAGAGATAATTAGAATTAATTTGACTGTAAACACAAAGATATTAGTACAAAATACGTGACGTAGAAAGTAATA

...............................................................................................

             3363                                                                           3444
Template     atttcttgggtagtttgcagttttaaaattatgttttaaaatggactatcatatgcttaccgtaacttgaaagtatttcgat
Clonal Se... ATTTCTTGGGTAGTTTGCAGTTTTAAAATTATGTTTTAAAATGGACTATCATATGCTTACCGTAACTTGAAAGTATTTCGAT

...............................................................................................

             3445                                                                           3526
Template     ttcttggctttatatatcttgtggaaaggacgaaacaccggtcatcttagtcattacctggttttagagctagaaatagcaa
Clonal Se... TTCTTGGCTTTATATATCTTGTGGAAAGGACGAAACACCGGTCATCTTAGTCATTACCTGGTTTTAGAGCTAGAAATAGCAA

...............................................................................................

             3527                                                                           3608
Template     gttaaaataaggctagtccgttatcaacttgaaaaagtggcaccgagtcggtgctttttttcatggtcatagctgtttccta
Clonal Se... GTTAAAATAAGGCTAGTCCGTTATCAACTTGAAAAAGTGGCACCGAGTCGGTGCTTTTTTTCATGGTCATAGCTGTTTCCTA

...............................................................................................
```

```
             3609                                                                         3690
Template     agggtaaaacgacggccagtgagggcctatttcccatgattccttcatatttgcatatacgatacaaggctgttagagagat
Clonal Se... AGGGTAAAACGACGGCCAGTGAGGGCCTATTTCCCATGATTCCTTCATATTTGCATATACGATACAAGGCTGTTAGAGAGAT

...............................................................................................

             3691                                                                         3772
Template     aattagaattaatttgactgtaaacacaaagatattagtacaaaatacgtgacgtagaaagtaataatttcttgggtagttt
Clonal Se... AATTAGAATTAATTTGACTGTAAACACAAAGATATTAGTACAAAATACGTGACGTAGAAAGTAATAATTTCTTGGGTAGTTT

...............................................................................................

             3773                                                                         3854
Template     gcagttttaaaattatgttttaaaatggactatcatatgcttaccgtaacttgaaagtatttcgatttcttggctttatata
Clonal Se... GCAGTTTTAAAATTATGTTTTAAAATGGACTATCATATGCTTACCGTAACTTGAAAGTATTTCGATTTCTTGGCTTTATATA

...............................................................................................

             3855                                                                         3936
Template     tcttgtggaaaggacgaaacaccgggcactgcggctggaggtgggttttagagctagaaatagcaagttaaaataaggctag
Clonal Se... TCTTGTGGAAAGGACGAAACACCGGGCACTGCGGCTGGAGGTGGGTTTTAGAGCTAGAAATAGCAAGTTAAAATAAGGCTAG

...............................................................................................

             3937                                                                         4018
Template     tccgttatcaacttgaaaaagtggcaccgagtcggtgctttttttcatggtcatagctgtttcctcatcgtaaaacgacggc
Clonal Se... TCCGTTATCAACTTGAAAAAGTGGCACCGAGTCGGTGCTTTTTTTCATGGTCATAGCTGTTTCCTCATCGTAAAACGACGGC

...............................................................................................

             4019                                                                         4100
Template     cagtgagggcctatttcccatgattccttcatatttgcatatacgatacaaggctgttagagagataattagaattaatttg
Clonal Se... CAGTGAGGGCCTATTTCCCATGATTCCTTCATATTTGCATATACGATACAAGGCTGTTAGAGAGATAATTAGAATTAATTTG

...............................................................................................

             4101                                                                         4182
Template     actgtaaacacaaagatattagtacaaaatacgtgacgtagaaagtaataatttcttgggtagtttgcagttttaaaattat
Clonal Se... ACTGTAAACACAAAGATATTAGTACAAAATACGTGACGTAGAAAGTAATAATTTCTTGGGTAGTTTGCAGTTTTAAAATTAT

...............................................................................................

             4183                                                                         4264
Template     gttttaaaatggactatcatatgcttaccgtaacttgaaagtatttcgatttcttggctttatatatcttgtggaaaggacg
Clonal Se... GTTTTAAAATGGACTATCATATGCTTACCGTAACTTGAAAGTATTTCGATTTCTTGGCTTTATATATCTTGTGGAAAGGACG

...............................................................................................

             4265                                                                         4346
Template     aaacaccgcacctacctaagaaccatccgttttagagctagaaatagcaagttaaaataaggctagtccgttatcaacttga
Clonal Se... AAACACCGCACCTACCTAAGAACCATCCGTTTTAGAGCTAGAAATAGCAAGTTAAAATAAGGCTAGTCCGTTATCAACTTGA

...............................................................................................

             4347                                                                         4428
Template     aaaagtggcaccgagtcggtgctttttttcatggtcatagctgtttcctacctgtaaaacgacggccagtgagggcctattt
Clonal Se... AAAAGTGGCACCGAGTCGGTGCTTTTTTTCATGGTCATAGCTGTTTCCTACCTGTAAAACGACGGCCAGTGAGGGCCTATTT

...............................................................................................

             4429                                                                         4510
Template     cccatgattccttcatatttgcatatacgatacaaggctgttagagagataattagaattaatttgactgtaaacacaaaga
Clonal Se... CCCATGATTCCTTCATATTTGCATATACGATACAAGGCTGTTAGAGAGATAATTAGAATTAATTTGACTGTAAACACAAAGA

...............................................................................................
```

```
             4511                                                                              4592
Template     tattagtacaaaatacgtgacgtagaaagtaataatttcttgggtagtttgcagttttaaaattatgttttaaaatggacta
Clonal Se... TATTAGTACAAAATACGTGACGTAGAAAGTAATAATTTCTTGGGTAGTTTGCAGTTTTAAAATTATGTTTTAAAATGGACTA

.................................................................................................

             4593                                                                              4674
Template     tcatatgcttaccgtaacttgaaagtatttcgatttcttggctttatatatcttgtggaaaggacgaaacaccgttcgtatc
Clonal Se... TCATATGCTTACCGTAACTTGAAAGTATTTCGATTTCTTGGCTTTATATATCTTGTGGAAAGGACGAAACACCGTTCGTATC

.................................................................................................

             4675                                                                              4756
Template     tgtaaaaccaaggttttagagctagaaatagcaagttaaaataaggctagtccgttatcaacttgaaaaagtggcaccgagt
Clonal Se... TGTAAAACCAAGGTTTTAGAGCTAGAAATAGCAAGTTAAAATAAGGCTAGTCCGTTATCAACTTGAAAAAGTGGCACCGAGT

.................................................................................................

             4757                                                                              4838
Template     cggtgctttttttcatggtcatagctgtttcctgcgagtaaaacgacggccagtgagggcctatttcccatgattccttcat
Clonal Se... CGGTGCTTTTTTTCATGGTCATAGCTGTTTCCTGCGAGTAAAACGACGGCCAGTGAGGGCCTATTTCCCATGATTCCTTCAT

.................................................................................................

             4839                                                                              4920
Template     atttgcatatacgatacaaggctgttagagagataattagaattaatttgactgtaaacacaaagatattagtacaaaatac
Clonal Se... ATTTGCATATACGATACAAGGCTGTTAGAGAGATAATTAGAATTAATTTGACTGTAAACACAAAGATATTAGTACAAAATAC

.................................................................................................

             4921                                                                              5002
Template     gtgacgtagaaagtaataatttcttgggtagtttgcagttttaaaattatgttttaaaatggactatcatatgcttaccgta
Clonal Se... GTGACGTAGAAAGTAATAATTTCTTGGGTAGTTTGCAGTTTTAAAATTATGTTTTAAAATGGACTATCATATGCTTACCGTA

.................................................................................................

             5003                                                                              5084
Template     acttgaaagtatttcgatttcttggctttatatatcttgtggaaaggacgaaacaccgcacggtcaccctgacacgctgttt
Clonal Se... ACTTGAAAGTATTTCGATTTCTTGGCTTTATATATCTTGTGGAAAGGACGAAACACCGCACGGTCACCCTGACACGCTGTTT

.................................................................................................

             5085                                                                              5166
Template     tagagctagaaatagcaagttaaaataaggctagtccgttatcaacttgaaaaagtggcaccgagtcggtgctttttttcat
Clonal Se... TAGAGCTAGAAATAGCAAGTTAAAATAAGGCTAGTCCGTTATCAACTTGAAAAAGTGGCACCGAGTCGGTGCTTTTTTTCAT

.................................................................................................

             5167                                                                              5248
Template     ggtcatagctgtttcctcgtagcaagcaagcgctcgaaacggtgcagcggctgttgccggtgctgtgccaggaccatggcct
Clonal Se... GGTCATAGCTGTTTCCTCGTAGCAAGCAAGCGCTCGAAACGGTGCAGCGGCTGTTGCCGGTGCTGTGCCAGGACCATGGCCT

.................................................................................................

             5249                                                                              5330
Template     gaccccggaccaagtggtggctatcgagacgtctagaccagccaggacagaaatgcctcgacttcgctgctacccaaggttg
Clonal Se... GACCCCGGACCAAGTGGTGGCTATCGAGACGTCTAGACCAGCCAGGACAGAAATGCCTCGACTTCGCTGCTACCCAAGGTTG

.................................................................................................

             5331                                                                              5412
Template     ccgggtgacgcacaccgtggaaacggatgaaggcacgaacccagtggacataagcctgttcggttcgtaagctgtaatgcaa
Clonal Se... CCGGGTGACGCACACCGTGGAAACGGATGAAGGCACGAACCCAGTGGACATAAGCCTGTTCGGTTCGTAAGCTGTAATGCAA

.................................................................................................
```

```
             5413                                                                           5494
Template     gtagcgtatgcgctcacgcaactggtccagaaccttgaccgaacgcagcggtggtaacggcgcagtggcggttttcatggct
Clonal Se... GTAGCGTATGCGCTCACGCAACTGGTCCAGAACCTTGACCGAACGCAGCGGTGGTAACGGCGCAGTGGCGGTTTTCATGGCT

...............................................................................................

             5495                                                                           5576
Template     tgttatgactgttttttggggtacagtctatgcctcgggcatccaagcagcaagcgcgttacgccgtgggtcgatgtttga
Clonal Se... TGTTATGACTGTTTTTTGGGGTACAGTCTATGCCTCGGGCATCCAAGCAGCAAGCGCGTTACGCCGTGGGTCGATGTTTGA

...............................................................................................

             5577                                                                           5658
Template     tgttatggagcagcaacgatgttacgcagcagggcagtcgccctaaaacaaagttaaacattatgagggaagcggtgatcgc
Clonal Se... TGTTATGGAGCAGCAACGATGTTACGCAGCAGGGCAGTCGCCCTAAAACAAAGTTAAACATTATGAGGGAAGCGGTGATCGC

...............................................................................................

             5659                                                                           5740
Template     cgaagtatcgactcaactatcagaggtagttggcgtcatcgagcgccatctcgaaccgacgttgctggccgtacatttgtac
Clonal Se... CGAAGTATCGACTCAACTATCAGAGGTAGTTGGCGTCATCGAGCGCCATCTCGAACCGACGTTGCTGGCCGTACATTTGTAC

...............................................................................................

             5741                                                                           5822
Template     ggctccgcagtggatggcggcctgaagccacacagtgatattgatttgctggttacggtgaccgtaaggcttgatgaaacaa
Clonal Se... GGCTCCGCAGTGGATGGCGGCCTGAAGCCACACAGTGATATTGATTTGCTGGTTACGGTGACCGTAAGGCTTGATGAAACAA

...............................................................................................

             5823                                                                           5904
Template     cgcggcgagctttgatcaacgaccttttggaaacttcggcttcccctggagagagcgagattctccgcgctgtagaagtcac
Clonal Se... CGCGGCGAGCTTTGATCAACGACCTTTTGGAAACTTCGGCTTCCCCTGGAGAGAGCGAGATTCTCCGCGCTGTAGAAGTCAC

...............................................................................................

             5905                                                                           5986
Template     cattgttgtgcacgacgacatcattccgtggcgttatccagctaagcgcgaactgcaatttggagaatggcagcgcaatgac
Clonal Se... CATTGTTGTGCACGACGACATCATTCCGTGGCGTTATCCAGCTAAGCGCGAACTGCAATTTGGAGAATGGCAGCGCAATGAC

...............................................................................................

             5987                                                                           6068
Template     attcttgcaggtatcttcgagccagccacgatcgacattgatctggctatcttgctgacaaaagcaagagaacatagcgttg
Clonal Se... ATTCTTGCAGGTATCTTCGAGCCAGCCACGATCGACATTGATCTGGCTATCTTGCTGACAAAAGCAAGAGAACATAGCGTTG

...............................................................................................

             6069                                                                           6150
Template     ccttggtaggtccagcggcggaggaactctttgatccggttcctgaacaggatctatttgaggcgctaaatgaaaccttaac
Clonal Se... CCTTGGTAGGTCCAGCGGCGGAGGAACTCTTTGATCCGGTTCCTGAACAGGATCTATTTGAGGCGCTAAATGAAACCTTAAC

...............................................................................................

             6151                                                                           6232
Template     gctatggaactcgccgcccgactgggctggcgatgagcgaaatgtagtgcttacgttgtcccgcatttggtacagcgcagta
Clonal Se... GCTATGGAACTCGCCGCCCGACTGGGCTGGCGATGAGCGAAATGTAGTGCTTACGTTGTCCCGCATTTGGTACAGCGCAGTA

...............................................................................................

             6233                                                                           6314
Template     accggcaaaatcgcgccgaaggatgtcgctgccgactgggcaatggagcgcctgccggcccagtatcagcccgtcatacttg
Clonal Se... ACCGGCAAAATCGCGCCGAAGGATGTCGCTGCCGACTGGGCAATGGAGCGCCTGCCGGCCCAGTATCAGCCCGTCATACTTG

...............................................................................................
```

```
              6315                                                                            6396
Template      aagctagacaggcttatcttggacaagaagaagatcgcttggcctcgcgcgcagatcagttggaagaatttgtccactacgt
Clonal Se...  AAGCTAGACAGGCTTATCTTGGACAAGAAGAAGATCGCTTGGCCTCGCGCGCAGATCAGTTGGAAGAATTTGTCCACTACGT

..........................................................................................

              6397                                                                            6478
Template      gaaaggcgagatcaccaaggtagtcggcaaataaccctcgagccacccatgaccaaaatcccttaacgtgagttacgcgtcg
Clonal Se...  GAAAGGCGAGATCACCAAGGTAGTCGGCAAATAACCCTCGAGCCACCCATGACCAAAATCCCTTAACGTGAGTTACGCGTCG

..........................................................................................

              6479                                        6522
Template      ttccactgagcgtcagaccccgtagaaaagatcaaaggatcttc
Clonal Se...  TTCCACTGAGCGTCAGACCCCGTAGAAAAGATCAAAGGATCTTC

..........................................................................................
```
